# Climate and local abundance in freshwater fishes

**DOI:** 10.1098/rsos.160093

**Published:** 2016-06-22

**Authors:** Jason H. Knouft, Melissa M. Anthony

**Affiliations:** 1 Department of Biology, Saint Louis University, 3507 Laclede Avenue, St Louis, Missouri 63103, USA; 2 Program in Integrated and Applied Sciences, Saint Louis University, 3507 Laclede Avenue, St Louis, Missouri 63103, USA

**Keywords:** abundant-centre, environmental niche, geographical information systems, population size, quantile regression

## Abstract

Identifying factors regulating variation in numbers of individuals among populations across a species' distribution is a fundamental goal in ecology. A common prediction, often referred to as the abundant-centre hypothesis, suggests that abundance is highest near the centre of a species' range. However, because of the primary focus on the geographical position of a population, this framework provides little insight into the environmental factors regulating local abundance. While range-wide variation in population abundance associated with environmental conditions has been investigated in terrestrial species, the relationship between climate and local abundance in freshwater taxa across species' distributions is not well understood. We used GIS-based temperature and precipitation data to determine the relationships between climatic conditions and range-wide variation in local abundance for 19 species of North American freshwater fishes. Climate predicted a portion of the variation in local abundance among populations for 18 species. In addition, the relationship between climatic conditions and local abundance varied among species, which is expected as lineages partition the environment across geographical space. The influence of local habitat quality on species persistence is well documented; however, our results also indicate the importance of climate in regulating population sizes across a species geographical range, even in aquatic taxa.

## 1. Introduction

Identification of the contemporary factors regulating local population abundance is a fundamental goal in ecology. Whittaker [1,2] provided formative evidence that variation in abundance within and among species is due to differential responses across habitat gradients, thus supporting the concept that species respond to environmental conditions based on tolerances to those conditions [3]. At larger spatial scales, studies of variation in population abundance have generally focused on documenting patterns across the geographical ranges of species (e.g. [4,5]). A common prediction, often referred to as the abundant-centre hypothesis, suggests that population abundance is highest near the centre of a species' geographical range and decreases towards the edge of the range [5–7]. Although the abundant-centre prediction has been described as a general rule in biogeography [4] and driven by a common feature shared by all species [8], this framework provides limited opportunities for direct investigation of environmental factors regulating local abundance because of the primary focus on the geographical position of a population (e.g. geographical coordinates or linear distance from the centre of the range). Hawkins & Diniz-Filho [9] recognized a similar limitation in studies that relate species richness to latitude and noted that hypotheses based on geographical positioning provide minimal benefits for advancing our understanding of spatial variation in biodiversity because of the lack of quantification of environmental conditions (or other spatially varying factors) across the region of interest.

While multiple mechanisms have been proposed to explain the abundant-centre distribution [6,7], from a niche-based perspective, the abundant-centre prediction implies that the most suitable habitat for a species is found near the centre of the geographical distribution and habitat quality decreases towards the edge of the species' range until the absence of suitable habitat contributes to the inability of populations to persist [3,5,10]. However, the quantification of multivariate habitat characteristics across the species range has historically been a challenge, thus limiting the broad-scale assessment of the environmental conditions regulating local population abundance. Fortunately, the availability of global environmental geographical information systems (GISs) data has recently allowed for the quantification of climatic aspects of the niche at broad spatial scales (i.e. the environmental niche) [11].

The term ‘environmental niche’ is used to refer to the estimation of the *n*-dimensional hypervolume described by Hutchinson [12] using integrated habitat and species distribution data, particularly at landscape and larger spatial scales [13,14]. Characterization of the environmental niche frequently excludes information on biotic interactions; however, this approach is useful for relating available habitat and species distributions and diversity at broad spatial scales [11]. Using this GIS-based approach, habitat gradients can be quantified across large geographical areas to assess the relationship between regional climatic conditions and variation in local abundance. If a significant component of variation in species abundance is predicted by broad-scale environmental factors such as climate, from a hierarchical perspective, these broad-scale variables should have a primary effect on abundance, while local habitat quality and biotic interactions generate secondary effects that regulate abundance under the constraints of the regional environmental conditions. In this scenario, regional climate defines the arena in which local interactions take place and optimal local habitat will maximize abundance only to the degree allowable by climatic conditions.

While the relationship between the environmental niche and range-wide variation in local population abundance has been investigated in terrestrial species [15], a taxonomically broad assessment of the relationship between climate and range-wide variation in local abundance in freshwater taxa is apparently not available. Nevertheless, the use of climatic variables in correlative approaches to predict aquatic species distributions is common (e.g. [16–18]), and based on the untested assumption that climate is a consistent predictor of local species persistence in aquatic systems across broad geographical regions. The goal of this research is to determine if there is a relationship between climatic conditions and local abundance in freshwater fishes. We use data from a standardized sampling methodology across the ranges of 19 North American freshwater fish species and GIS-based climate data to test the hypothesis that a portion of the variation in local population abundance in freshwater taxa can be predicted with broad-scale environmental data.

## 2. Material and methods

### 2.1. Fish data

Fish abundance data were assembled from the United States Geological Survey (USGS) National Water-Quality Assessment (NAWQA) program (http://water.usgs.gov/nawqa) [19–21]. The NAWQA dataset contains data derived from over 15 000 fish, invertebrate and algae samples from 51 river basins across the United States and has been used to assess, for example, the relationship between land use and species assemblage structure [22], impacts of non-native species on native species diversity [23] and relationships between species distributions, body size and local abundance [24]. NAWQA sites were generally sampled during low-flow seasons based on standardized stream section length and geomorphological characteristics using electrofishing and beach seining methods to ensure a representative sample of the fish assemblage [25,26]. Individuals were identified and counted at each locality to provide estimates of local abundance [21].

The NAWQA dataset is limited to sites within the United States. To effectively examine range-wide variation in local abundance, only non-migratory species with distributions contained entirely within the United States were used for this study. To ensure robust statistical analysis, species were also excluded if they occurred at less than 20 localities in the NAWQA dataset and did not cover the full extent of the species' geographical distribution. The region of North America north of Mexico contains approximately 828 species of native freshwater fishes [27]. A total of 19 species, representing eight families, were available for analyses after the geographical and sample size constraints were imposed on the NAWQA dataset (table 1).

**Table 1.** Results and variable coefficients from models predicting variation in local abundance for each species. ‘*N*’ indicates the number of sites for each species. AICc is the Akaike's information criterion score corrected for small sample size. $R_{\left(\tau \right)}^{1}$ is the pseudo-R^2^ statistic. s.e. is standard error. ‘—’ indicates cases where climate does not explain a greater portion of variation in local abundance over the mean based on the ΔAICc > 2 criterion.

species (*N*)							
family	AICc	$R_{\left(\tau \right)}^{1}$	variable	coefficient	s.e.	*t*_stat_	*p*-value
*Campostoma anomalum* (151)	265.42	0.146	constant	2.124	0.031	68.784	0.000
Cyprinidae			PC2	−0.199	0.026	−7.574	0.000
			PC1^2^	−0.229	0.021	−10.749	0.000
			PC1 × PC2	−0.170	0.022	−7.568	0.000
*Campostoma oligolepis* (84)	120.58	0.157	constant	1.991	0.038	52.582	0.000
Cyprinidae			PC1	0.191	0.068	2.817	0.006
			PC2^2^	−0.051	0.005	−9.888	0.000
*Cottus carolinae* (88)	171.45	0.090	constant	1.777	0.083	21.420	0.000
Cottidae			PC2^2^	−0.077	0.025	−3.111	0.003
			PC2 × PC3	0.134	0.044	3.058	0.003
*Cyprinella analostoma* (25)	18.30	0.629	constant	1.577	0.060	26.165	0.000
Cyprinidae			PC1	0.467	0.035	13.253	0.000
			PC2	0.228	0.035	6.462	0.000
			PC1^2^	−0.100	0.056	−1.792	0.088
*Cyprinella spiloptera* (117)	218.13	0.153	constant	1.748	0.060	29.258	0.000
Cyprinidae			PC1	−0.227	0.078	−2.905	0.004
			PC2	−0.110	0.094	−1.170	0.244
			PC1^2^	−0.128	0.027	−4.695	0.000
			PC1 × PC2	0.192	0.059	3.266	0.001
*Cyprinella venusta* (43)	86.09	0.113	constant	1.940	0.067	28.786	0.000
Cyprinidae			PC2	−0.161	0.068	−2.359	0.023
*Esox americanus* (44)	100.48	0.150	constant	1.805	0.200	9.000	0.000
Esocidae			PC1^2^	−0.454	0.116	−2.918	0.006
*Etheostoma blennioides* (102)	147.40	0.259	constant	1.195	0.079	15.145	0.000
Percidae			PC1	−0.324	0.074	−4.375	0.000
			PC1 × PC2	0.210	0.068	3.072	0.003
			PC2^2^	−0.097	0.051	−1.920	0.058
*Etheostoma caeruleum* (55)	103.46	0.074	constant	1.449	0.123	11.798	0.000
Percidae			PC1	0.114	0.124	0.920	0.362
*Etheostoma flabellare* (61)	99.87	0.186	constant	1.452	0.099	14.720	0.000
Percidae			PC1^2^	−0.144	0.046	−3.118	0.003
			PC1 × PC2	−0.085	0.088	−0.974	0.334
			PC2^2^	0.054	0.058	0.925	0.359
*Etheostoma olmstedi* (69)	116.86	0.163	constant	1.564	0.061	25.561	0.000
Percidae			PC1	−0.217	0.062	−3.524	0.001
			PC2	0.182	0.062	2.956	0.004
*Fundulus olivaceus* (39)	22.05	0.509	constant	1.148	0.073	15.640	0.000
Fundulidae			PC2	−0.217	0.041	−5.287	0.000
			PC2^2^	−0.344	0.057	−5.991	0.000
			PC3	0.204	0.039	5.183	0.000
			PC3^2^	−0.052	0.019	−2.793	0.009
*Lepistoseus oculatus* (36)	27.66	0.225	constant	0.888	0.048	18.448	0.000
Lepisosteidae			PC1	0.181	0.048	3.736	0.001
			PC2^2^	−0.071	0.017	−4.297	0.000
*Lepistoseus osseus* (24)	18.27	0.555	constant	1.200	0.064	18.732	0.000
Lepisosteidae			PC1	0.414	0.045	9.271	0.000
			PC2	−0.101	0.040	−2.541	0.019
			PC2^2^	−0.520	0.053	−9.756	0.000
*Moxostoma duquesnei* (80)	116.45	0.071	constant	1.087	0.067	15.805	0.000
Catostomidae			PC2	−0.051	0.058	0.881	0.381
			PC2^2^	−0.085	0.044	−1.951	0.055
*Moxostoma erythrurum* (100)	154.40	0.140	constant	1.042	0.078	13.392	0.000
Catostomidae			PC1	−0.101	0.078	−1.293	0.199
			PC2	−0.157	0.078	−2.013	0.047
*Noturus exilis* (20)	24.94	0.435	constant	1.271	0.078	16.192	0.000
Ictaluridae			PC1	0.481	0.104	4.629	0.000
			PC1^2^	-0.413	0.103	−4.015	0.001
			PC2^2^	0.057	0.065	0.868	0.398
*Percina nigrofasciata* (39)							
Percidae	—	—	—	—	—	—	—
*Percina sciera* (22)	0.08	0.738	constant	0.407	0.030	13.427	0.000
Percidae			PC1	0.480	0.023	20.982	0.000
			PC1 × PC2	0.085	0.023	3.725	0.002
			PC1^2^	0.111	0.024	4.648	0.000

Although NAWQA samples were based on a standardized sampling methodology, peculiarities associated with site-specific sampling may influence abundance estimates. Rarefaction was used in this study to account for the influence of potential sampling bias on estimates of local population abundance [28]. Separate rarefaction estimates were generated for each of the 19 species. The sample size during rarefaction resampling was based on the total number of individuals (of all species) at the site where the least number of total individuals were collected [23]. For example, if a species occurred at 50 sites and the total number of individuals of all species at each site ranged from 100 to 1000, then 100 individuals were resampled from each of the 50 sites during rarefaction. Data from each site were resampled 1000 times without replacement to generate rarefied estimates of local abundance for each species occurring at the site (EcoSim, v. 7.0, [29]) [23]. This process resulted in rarefied estimates of local population abundance for the focal species.

### 2.2. Climate variables

Data for 10 climate variables were assembled from the Worldclim dataset (www.worldclim.org) ([30]; table 2). Owing to the covariation of climate variables, climate data were subjected to a principal components analysis (PCA) for each species to produce a set of uncorrelated variables. All climate data, except temperature and precipitation seasonality, were log_10_-transformed prior to statistical modelling and separate analyses were carried out for each of the 19 fish species. Principal components with eigenvalues greater than 1.0 were retained for further analyses, resulting in two or three principal components with loadings that varied among species (electronic supplementary material, appendix SA).

**Table 2.** Climate variables used in all analyses.

climate variable
mean annual temperature (K)
mean diurnal range (K) [*mean of monthly* (*maximum temperature – minimum temperature*)]
temperature seasonality (*standard deviation of monthly mean temperatures*)
maximum temperature of warmest month (K)
minimum temperature of coldest month (K)
temperature annual range (K)
annual precipitation (mm)
precipitation of wettest month (mm)
precipitation of driest month (mm)
precipitation seasonality (*coefficient of variation of monthly precipitation measures*)

### 2.3. Modelling individual species using linear quantile regression

The relationship between climate and log_10_(abundance) was examined using linear quantile regression. Quantile regression has been demonstrated to be preferable to ordinary least-squares (OLS) regression for analysing ecological data in cases where the limits of species abundance are a result of complex interactions between measured and unmeasured variables [31]. In this study, climate data are the measured variables and local habitat availability and biotic interactions are the unmeasured variables. In typical OLS regression, the effect of a set of predictors on the rate of change of the response is determined for the conditional mean of the response distribution. The conditional mean is the portion of the response distribution where the interactions between predictors of interest and other unmeasured predictors are most likely to occur. However, determining the rate of change of the conditional response near the upper boundary of its distribution has been demonstrated to provide a more accurate estimate of the limiting effect of measured predictors where they are most likely to be the primary factor limiting abundance [31]. A quantile regression approach can model any portion of the conditional response distribution, referred to as quantile (*τ*), which takes on values from 0 to 1. The model uses an optimization function that minimizes the sum of asymmetrically weighted absolute residuals based on the value of *τ* (for a more in-depth treatment of modelling using regression quantiles, see [31,32]). The limiting effect of climate for each fish species was modelled at the 95th percentile of the abundance, corresponding to *τ *= 0.95 (similar to [33]).

### 2.4. Model selection

Final models for each species were determined using stepwise variable selection. Along with the main effect of each principal component, quadratic terms and interaction terms were also considered for inclusion in the final model to account for any curvilinear or synergistic effects. The retention of variables for the final model at each step of selection was determined using Akaike Information Criterion for small sample sizes (AICc). AICc is calculated using the negative log likelihood of the model, a penalty for including additional variables into the model to avoid over-fitting, and a bias correction term for small sample size, which results in an overall measure of lack of model fit to the data [34]. Stepwise selection began with a model containing only the intercept term. Single predictor variables were then added to the model and the variable generating the largest improvement based on the ΔAICc measure was retained. The cut-off for variable inclusion was an improvement in AICc of greater than two (ΔAICc > 2) [34]. After the addition of each variable, variables already in the model were re-examined for inclusion because variables entered in a previous step may become superfluous at later steps due to their relationship with other variables [35]. This was done by deleting individual variables from the model and removing variables which no longer contributed to significant improvement of AICc (i.e. ΔAICc > 2). This process was repeated until the addition or removal of variables no longer improved the model, resulting in the final model for each species. We chose a stepwise model selection approach as opposed to comparing the full model to candidate models based on *a priori* hypotheses for multiple reasons. First, the untested inclusion of interaction and polynomial terms in a full model results in an extremely complicated model with terms that are difficult to interpret. Moreover, the inclusion of interaction terms is dependent on the importance of the main effect of the interacting variables, thus the inclusion of interaction terms in *a priori* models can be overly speculative and challenging to conceptualize. Finally, the use of species-specific PCA produces univariate variables with varying characteristics for each taxon and makes the generation of consistent *a priori* hypotheses impossible.

Standard errors for parameters were calculated using the Markov chain marginal bootstrap (MCMB) developed by He & Hu [36]. For species with sample sizes less than 50, the sparsity method of confidence interval estimation was used because resampling methods become unstable at small sample sizes [37]. Goodness of fit of the final model for each species was determined using the $R_{\left(\tau \right)}^{1}$ statistic proposed in [38]. While traditional *R*^2^ measures model performance on the conditional mean response, $R_{\left(\tau \right)}^{1}$ is a local measure of fit that characterizes model performance on the specified quantile of the response distribution [15]. $R_{\left(\tau \right)}^{1}$ is calculated for the specified quantile by first dividing the residuals of the full model by the residuals of the intercept-only (null) model. This quantity is then subtracted from 1.0, yielding a quantity interpretable as the per cent reduction in error obtained using the full model as opposed to the null model [38]. All analyses were carried out using the quantreg package [39] in R v. 3.1 software [40].

## 3. Results

**Figure 1.**
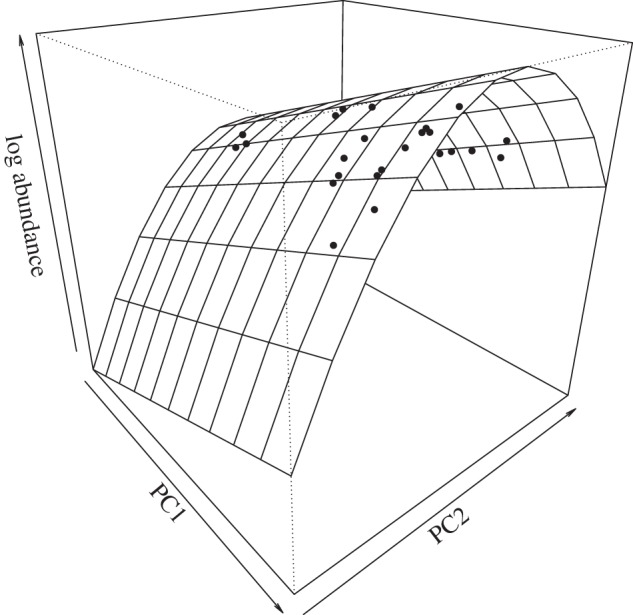
Relationship between principal component (PC) scores describing temperature and precipitation and log_10_(abundance) in the longnose gar (*Lepisosteus osseus*).

Eighteen of 19 species models include variables that predict abundance data better than random (i.e. better than a model with only a constant = 0) using the ΔAICc (table 1). $R_{\left(\tau \right)}^{1}$ values among the final models ranged from 0.071 to 0.738 with an average of 0.240 among models (table 1). Species with smaller sample sizes tended to have larger $R_{\left(\tau \right)}^{1}$ values (*R*^2^ = 0.363, *p* = 0.001), suggesting that the limited density of localities for certain species did not inhibit our ability to detect relationships among variables. In addition, the model selection approach and the limited number of predictor variables in each model suggest that model overfitting is probably not driving this relationship. A graphical example of the relationship between climate and local abundance for the longnose gar (*Lepisosteus osseus*) is presented in figure 1.

Based on principal component scores and loadings, temperature is positively correlated with local abundance in *Cyprinella analostoma*, *L. osseus*, *L. oculatus*, *Campostoma oligolepis*, *Cy. venusta* and *Etheostoma caeruleum*, and negatively correlated with local abundance in *E. blennioides*, *E. olmstedi* and *Moxostoma erythrurum*. Precipitation is positively correlated with local abundance in *Cy. analostoma* and *Ca. oligolepis* and negatively correlated with local abundance in *L. osseus*, *E. blennioides*, *E. olmstedi*, *Ca. anomalum*, and *M. erythrurum*. Fourteen of the 19 final models contained one or more squared terms, which indicates that local abundance is maximized at intermediate values of climate (table 1). Six of the 19 final models contained an interaction term, which indicates the strength of the association between particular climate variables and local abundance changes as a function of other climate variables (table 1).

## 4. Discussion

Recent advances in the availability of GIS-based global environmental data have afforded the opportunity to examine variation in population sizes across a species' distribution, beyond simply relating the size of a population to its position in the landscape. In particular, estimation of a climate-based environmental niche for a species provides the ability to examine variation in population size in the context of apparent species tolerances to environmental gradients. While relating population sizes to environmental gradients has been widely investigated in terrestrial taxa (e.g. [2]), this relationship has received less attention in freshwater species and generally focused on limited geographical areas [41–44], possibly due to limited amounts of standardized abundance data. Results from our niche-based approach using GIS environmental data support the hypothesis that climate is responsible for some aspect of local abundance in freshwater taxa, with climate variables explaining a portion of the variation in abundance in 18 of 19 species examined. While our quantile regression approach acknowledges the importance of local habitat and biotic interactions in the regulation of population size, results support the prediction that climate is important to population-level processes and not only at the edge of a species' distribution, but also throughout the entire range. Dispersal and stream network geometry are unlikely to influence variation in population sizes at our study scale due to the relatively limited intra-annual dispersal distances in most non-migratory North American fish species. However, these spatial influences are potentially important at smaller (e.g. watershed) scales.

The relationships between principal component (PC) climate variables and local abundance are different among species, which is expected as lineages partition the environment across geographical space [45]. Many species in this study exhibit linear relationships between climate and abundance. In particular, eight species exhibit either positive or negative correlations with temperature (based on principal component scores and loadings), suggesting that population abundance tends to peak near the warmest or coldest parts of the species' range. In these cases, physical features such as watershed boundaries may constrain species distributions as opposed to climatic conditions, particularly in warmer areas. For example, several species reach their highest abundances near the Gulf of Mexico (*Cy. venusta*, *L. oculatus*, *L. osseus*), which is an obvious barrier to expansion into warmer regions. Variation in abundance in 14 species is predicted by at least one squared variable, indicating a Gaussian-type relationship between local abundance and climate [46]. This suggests that particular aspects of climate may be more important in regulating these species' distributions than physical attributes of the landscape as species abundance peaks at an intermediate point along the species' perceived climatic range.

*Percina nigrofasciata* is the only species that does not exhibit a relationship between climate and local abundance. Other than Type II error, this lack of relationship may be a result of physical constraints on the species' distribution. The majority of the species range, which is relatively small compared with other species in this dataset, occurs in watersheds that drain directly to the Gulf of Mexico (e.g. the Mobile River basin) [27]. Thus, the environmental tolerances of the species may not be fully realized by the species due to northern headwater and southern Gulf of Mexico distributional constraints. These constraints may limit detection of the response of abundance to the full climatic tolerances of the species.

The translation of climate to aquatic environmental conditions is a complex process and generally realized as hydrological regimes and water temperature. While flow, seasonal variability in flow and water temperature directly affect aquatic taxa and estimates of these variables would probably enhance our analyses, the generation of these types of data to address ecological patterns is constrained by available stream gauge monitoring (e.g. [47]) or requires a landscape-scale hydrological modelling approach [48,49]. However, prediction of local flow and water temperatures across a continental scale using this approach is computationally prohibitive; therefore, the application of non-aquatic data surrogates (such as climate) is necessary. Air temperature and water temperature are correlated at broad spatial scales [50,51], and precipitation and air temperature can serve as predictors of river flows due to their determinant roles in the hydrological cycle (e.g. rainfall contribution and evapotranspiration), at least at continental scales. However, the ability of climate variables to explain a portion of the variation in local abundance of aquatic taxa across the range of a species has not been addressed. Results from this study provide support that climate data can explain the distribution of freshwater taxa at broad spatial scales and improve the ability to predict local population sizes.

The abundant-centre hypothesis provides little more than a framework for speculation regarding factors that regulate population sizes over broad geographical areas and should be discarded in favour of more biologically meaningful hypotheses. Our results indicate that climate not only explains variation in abundance of aquatic species across their geographical range, but species vary in their response to gradients in climate in ways that are not consistent with predictions from the abundant-centre hypothesis. In particular, the abundant-centre hypothesis fails to consider physical aspects of the landscape in limiting the distribution of species, as well as the location of climatic optima, which can potentially occur in any portion of a species' geographical range. The differential response of species to temperature and precipitation along their geographical range indicates that climate is a meaningful predictor of local abundance of freshwater fishes. This, along with increasingly available GIS-based data resources on other biologically relevant environmental conditions will further improve the current understanding of factors regulating local abundance of freshwater species.

## Supplementary Material

Appendix A. Principal component loadings for climate variables for each species.

## Data accessibility

Supporting data for this study can be accessed at http://dx.doi.org/10.5061/dryad.1d1t5.
