# Supplementary material for: Climate and local abundance in freshwater fishes
Source: R Soc Open Sci. 2016 Jun 22;3(6):160093. doi: 10.1098/rsos.160093 (PMC4929904; doi:10.1098/rsos.160093)
Supplement: Appendix A. Principal component loadings for climate variables for each species. [file rsos160093supp1.pdf]

Appendix A. Principal component loadings for climate variables for each species.

Species	Climate Variable	PC1	PC2	PC3
Campostoma anomalum	Mean Annual Temperature (K)	0.938	0.275	-
	Mean Diurnal Range (K)	0.581	0.257	-
	Temperature Seasonality	-0.817	-0.507	-
	Maximum Temperature of Warmest Month (K)	0.828	-0.333	-
	Minimum Temperature of Coldest Month (K)	0.917	0.362	-
	Temperature Annual Range (K)	-0.744	-0.585	-
	Annual Precipitation (mm)	0.154	0.937	-
	Precipitation of Wettest Month (mm)	0.096	0.679	-
	Precipitation of Driest Month (mm)	0.342	0.913	-
	Precipitation Seasonality	-0.256	-0.898	-
Campostoma oligolepis	Mean Annual Temperature (K)	0.862	0.443	-
	Mean Diurnal Range (K)	-0.003	0.943	-
	Temperature Seasonality	-0.961	-0.158	-
	Maximum Temperature of Warmest Month (K)	0.453	0.743	-

	Minimum Temperature of Coldest Month (K)	0.957	0.210	-
	Temperature Annual Range (K)	-0.975	0.105	-
	Annual Precipitation (mm)	0.926	0.275	-
	Precipitation of Wettest Month (mm)	0.843	0.165	-
	Precipitation of Driest Month (mm)	0.897	0.278	-
	Precipitation Seasonality	-0.666	-0.253	-
Cottus carolinae	Mean Annual Temperature (K)	0.382	0.849	-0.287
	Mean Diurnal Range (K)	0.008	-0.127	0.860
	Temperature Seasonality	-0.686	0.136	0.602
	Maximum Temperature of Warmest Month (K)	-0.004	0.968	0.135
	Minimum Temperature of Coldest Month (K)	0.598	0.488	-0.589
	Temperature Annual Range (K)	-0.642	0.185	0.722
	Annual Precipitation (mm)	0.811	0.517	-0.121
	Precipitation of Wettest Month (mm)	0.654	0.684	-0.212
	Precipitation of Driest Month (mm)	0.939	-0.124	-0.056
	Precipitation Seasonality	-0.256	0.927	-0.011

Cyprinella analostoma	Mean Annual Temperature (K)	0.972	0.066	-
	Mean Diurnal Range (K)	0.722	0.339	-
	Temperature Seasonality	-0.968	-0.226	-
	Maximum Temperature of Warmest Month (K)	0.946	0.099	-
	Minimum Temperature of Coldest Month (K)	0.978	0.095	-
	Temperature Annual Range (K)	-0.964	-0.096	-
	Annual Precipitation (mm)	0.427	0.822	-
	Precipitation of Wettest Month (mm)	0.784	0.491	-
	Precipitation of Driest Month (mm)	-0.093	0.887	-
	Precipitation Seasonality	0.924	0.117	-
Cyprinella spiloptera	Mean Annual Temperature (K)	0.889	0.386	-
	Mean Diurnal Range (K)	0.132	0.769	-
	Temperature Seasonality	-0.897	-0.381	-
	Maximum Temperature of Warmest Month (K)	0.586	0.639	-
	Minimum Temperature of Coldest Month (K)	0.937	0.335	-
	Temperature Annual Range (K)	-0.956	-0.255	-

	Annual Precipitation (mm)	0.822	0.490	-
	Precipitation of Wettest Month (mm)	0.292	0.833	-
	Precipitation of Driest Month (mm)	0.947	0.276	-
	Precipitation Seasonality	-0.954	-0.124	-
Cyprinella venusta	Mean Annual Temperature (K)	-0.497	0.821	-
	Mean Diurnal Range (K)	-0.044	0.694	-
	Temperature Seasonality	0.101	-0.986	-
	Maximum Temperature of Warmest Month (K)	-0.818	0.197	-
	Minimum Temperature of Coldest Month (K)	-0.296	0.926	-
	Temperature Annual Range (K)	-0.087	-0.959	-
	Annual Precipitation (mm)	0.979	-0.048	-
	Precipitation of Wettest Month (mm)	0.922	0.113	-
	Precipitation of Driest Month (mm)	0.927	-0.232	-
	Precipitation Seasonality	-0.849	0.258	-
Esox americanus	Mean Annual Temperature (K)	0.978	0.003	-
	Mean Diurnal Range (K)	0.666	0.027	-

	Temperature Seasonality	-0.938	-0.116	-
	Maximum Temperature of Warmest Month (K)	0.901	-0.080	-
	Minimum Temperature of Coldest Month (K)	0.972	0.080	-
	Temperature Annual Range (K)	-0.882	-0.136	-
	Annual Precipitation (mm)	0.705	0.599	-
	Precipitation of Wettest Month (mm)	0.857	0.177	-
	Precipitation of Driest Month (mm)	0.368	0.908	-
	Precipitation Seasonality	0.338	-0.876	-
Etheostoma blennioides	Mean Annual Temperature (K)	0.964	-0.088	-
	Mean Diurnal Range (K)	0.649	-0.347	-
	Temperature Seasonality	-0.585	0.747	-
	Maximum Temperature of Warmest Month (K)	0.849	0.428	-
	Minimum Temperature of Coldest Month (K)	0.880	-0.334	-
	Temperature Annual Range (K)	-0.352	0.842	-
	Annual Precipitation (mm)	0.761	-0.436	-
	Precipitation of Wettest Month (mm)	0.818	-0.258	-

	Precipitation of Driest Month (mm)	0.460	-0.773	-
	Precipitation Seasonality	0.224	0.866	-
Etheostoma caeruleum	Mean Annual Temperature (K)	0.657	0.713	0.201
	Mean Diurnal Range (K)	0.114	0.942	-0.268
	Temperature Seasonality	-0.834	-0.321	0.327
	Maximum Temperature of Warmest Month (K)	0.238	0.859	0.429
	Minimum Temperature of Coldest Month (K)	0.900	0.367	0.077
	Temperature Annual Range (K)	-0.882	0.297	0.281
	Annual Precipitation (mm)	0.894	0.331	-0.014
	Precipitation of Wettest Month (mm)	0.920	0.295	0.062
	Precipitation of Driest Month (mm)	0.858	0.125	-0.408
	Precipitation Seasonality	-0.181	0.052	0.966
Etheostoma flabellare	Mean Annual Temperature (K)	0.424	0.876	-
	Mean Diurnal Range (K)	0.560	0.649	-
	Temperature Seasonality	-0.906	-0.356	-
	Maximum Temperature of Warmest Month (K)	-0.233	0.946	-

	Minimum Temperature of Coldest Month (K)	0.684	0.683	-
	Temperature Annual Range (K)	-0.946	-0.197	-
	Annual Precipitation (mm)	0.711	0.536	-
	Precipitation of Wettest Month (mm)	0.438	0.619	-
	Precipitation of Driest Month (mm)	0.934	0.285	-
	Precipitation Seasonality	-0.956	0.014	-
Etheostoma olmstedi	Mean Annual Temperature (K)	0.963	0.099	-
	Mean Diurnal Range (K)	0.672	-0.205	-
	Temperature Seasonality	-0.968	-0.187	-
	Maximum Temperature of Warmest Month (K)	0.950	-0.076	-
	Minimum Temperature of Coldest Month (K)	0.964	0.153	-
	Temperature Annual Range (K)	-0.918	-0.290	-
	Annual Precipitation (mm)	0.415	0.859	-
	Precipitation of Wettest Month (mm)	0.820	0.461	-
	Precipitation of Driest Month (mm)	-0.303	0.899	-
	Precipitation Seasonality	0.897	-0.214	-

Fundulus olivaceus	Mean Annual Temperature (K)	0.954	-0.287	0.061
	Mean Diurnal Range (K)	0.032	0.815	-0.145
	Temperature Seasonality	-0.914	-0.295	-0.167
	Maximum Temperature of Warmest Month (K)	0.374	-0.832	-0.215
	Minimum Temperature of Coldest Month (K)	0.981	-0.113	0.146
	Temperature Annual Range (K)	-0.947	-0.145	-0.234
	Annual Precipitation (mm)	0.596	0.603	0.456
	Precipitation of Wettest Month (mm)	0.548	0.721	0.302
	Precipitation of Driest Month (mm)	0.214	0.159	0.872
	Precipitation Seasonality	-0.103	0.031	-0.817
Lepistoseus oculatus	Mean Annual Temperature (K)	0.972	0.045	-
	Mean Diurnal Range (K)	0.532	-0.135	-
	Temperature Seasonality	-0.923	-0.169	-
	Maximum Temperature of Warmest Month (K)	0.750	-0.266	-
	Minimum Temperature of Coldest Month (K)	0.973	0.152	-
	Temperature Annual Range (K)	-0.816	-0.358	-

	Annual Precipitation (mm)	-0.020	0.968	-
	Precipitation of Wettest Month (mm)	0.183	0.861	-
	Precipitation of Driest Month (mm)	0.019	0.943	-
	Precipitation Seasonality	0.066	-0.817	-
Lepistoseus osseus	Mean Annual Temperature (K)	0.984	-0.019	-
	Mean Diurnal Range (K)	0.745	0.210	-
	Temperature Seasonality	-0.940	-0.189	-
	Maximum Temperature of Warmest Month (K)	0.852	-0.304	-
	Minimum Temperature of Coldest Month (K)	0.983	0.060	-
	Temperature Annual Range (K)	-0.854	-0.313	-
	Annual Precipitation (mm)	0.019	0.975	-
	Precipitation of Wettest Month (mm)	0.352	0.820	-
	Precipitation of Driest Month (mm)	0.192	0.941	-
	Precipitation Seasonality	0.152	-0.858	-
Moxostoma duquesnei	Mean Annual Temperature (K)	0.920	-0.308	-
	Mean Diurnal Range (K)	0.580	-0.525	-

	Temperature Seasonality	-0.454	0.853	-
	Maximum Temperature of Warmest Month (K)	0.941	0.223	-
	Minimum Temperature of Coldest Month (K)	0.783	-0.539	-
	Temperature Annual Range (K)	-0.271	0.877	-
	Annual Precipitation (mm)	0.834	-0.418	-
	Precipitation of Wettest Month (mm)	0.914	-0.217	-
	Precipitation of Driest Month (mm)	0.457	-0.761	-
	Precipitation Seasonality	0.232	0.928	-
Moxostoma erythrurum	Mean Annual Temperature (K)	0.822	0.500	-
	Mean Diurnal Range (K)	0.174	0.768	-
	Temperature Seasonality	-0.882	-0.425	-
	Maximum Temperature of Warmest Month (K)	0.403	0.769	-
	Minimum Temperature of Coldest Month (K)	0.892	0.434	-
	Temperature Annual Range (K)	-0.948	-0.288	-
	Annual Precipitation (mm)	0.789	0.549	-
	Precipitation of Wettest Month (mm)	0.329	0.803	-

	Precipitation of Driest Month (mm)	0.931	0.334	-
	Precipitation Seasonality	-0.954	-0.175	-
Noturus exilis	Mean Annual Temperature (K)	0.671	0.696	-
	Mean Diurnal Range (K)	0.030	0.932	-
	Temperature Seasonality	-0.816	-0.572	-
	Maximum Temperature of Warmest Month (K)	0.266	0.867	-
	Minimum Temperature of Coldest Month (K)	0.812	0.550	-
	Temperature Annual Range (K)	-0.951	-0.296	-
	Annual Precipitation (mm)	0.914	0.372	-
	Precipitation of Wettest Month (mm)	0.977	-0.122	-
	Precipitation of Driest Month (mm)	0.820	0.547	-
	Precipitation Seasonality	-0.600	-0.728	-
Percina nigrofasciata	Mean Annual Temperature (K)	0.806	0.550	-
	Mean Diurnal Range (K)	0.009	0.604	-
	Temperature Seasonality	-0.936	-0.085	-
	Maximum Temperature of Warmest Month (K)	0.515	0.759	-

	Minimum Temperature of Coldest Month (K)	0.905	0.403	-
	Temperature Annual Range (K)	-0.949	0.108	-
	Annual Precipitation (mm)	-0.162	-0.891	-
	Precipitation of Wettest Month (mm)	-0.063	-0.863	-
	Precipitation of Driest Month (mm)	-0.591	-0.774	-
	Precipitation Seasonality	0.632	0.607	-
Percina sciera	Mean Annual Temperature (K)	0.985	-0.033	-
	Mean Diurnal Range (K)	0.595	0.488	-
	Temperature Seasonality	-0.890	-0.368	-
	Maximum Temperature of Warmest Month (K)	0.877	-0.350	-
	Minimum Temperature of Coldest Month (K)	0.991	0.068	-
	Temperature Annual Range (K)	-0.824	-0.459	-
	Annual Precipitation (mm)	0.055	0.958	-
	Precipitation of Wettest Month (mm)	0.462	0.766	-
	Precipitation of Driest Month (mm)	0.110	0.960	-
	Precipitation Seasonality	0.121	-0.915	-
